# Effects of Clinical Trial or Research Program Participation Status on In‐Hospital Mortality After Transcatheter Aortic Valve Implantation

**DOI:** 10.1161/JAHA.121.025920

**Published:** 2022-06-14

**Authors:** Tadao Aikawa, Toshiki Kuno, Jef Van den Eynde, Alexandros Briasoulis, Aaqib H. Malik

**Affiliations:** ^1^ Department of Cardiology Hokkaido Cardiovascular Hospital Sapporo Japan; ^2^ Department of Radiology Jichi Medical University Saitama Medical Center Saitama Japan; ^3^ Division of Cardiology, Montefiore Medical Center Albert Einstein College of Medicine New York NY; ^4^ Helen B. Taussig Heart Center The Johns Hopkins Hospital and School of Medicine Baltimore MD; ^5^ Department of Cardiovascular Sciences KU Leuven Leuven Belgium; ^6^ Section of Heart Failure and Transplantation, Division of Cardiovascular Medicine University of Iowa Iowa City IA; ^7^ Department of Cardiology Westchester Medical Center Valhalla NY

**Keywords:** clinical research participation, in‐hospital mortality, transcatheter aortic valve implantation, Valvular Heart Disease, Aortic Valve Replacement/Transcather Aortic Valve Implantation, Mortality/Survival

The development of novel devices and the favorable results of several randomized clinical trials have allowed for the rapid expansion of transcatheter aortic valve implantation (TAVI) to elderly patients with aortic stenosis across all risk categories^1^; however, the highly selected populations that are typically enrolled in randomized clinical trials may limit generalizability of the results to the real‐world population with aortic stenosis. Furthermore, clinical trial or research program participation itself can facilitate behavior change in patients and health care providers and may contribute to improved patient outcomes, which is known as the “Hawthorne effect.”^2^ Previous studies reported that research participation was associated with better survival in patients with acute coronary syndrome.^3^, ^4^ Given the lack of data exploring the effect of research participation on outcomes after TAVI, we compared the short‐term survival after TAVI between clinical research participants and nonparticipants using the Nationwide Inpatient Sample.

The data that support the findings of this study are available from the corresponding author upon reasonable request. The Nationwide Inpatient Sample is the largest publicly available all‐payer inpatient health care database in the United States and did not require ethical approval. All patients who underwent TAVI between 2013 and 2019 (n=56 648) were identified from the Nationwide Inpatient Sample using the following *International Classification of Diseases, Tenth Revision, Clinical Modification (ICD‐10‐CM)* codes: 02RF37H, 02RF37Z, 02RF38H, 02RF38Z, 02RF3JH, 02RF3JZ, 02RF3KH, and 02RF3KZ. Patients with age ≤18 years (n=22), cirrhosis (n=760), end‐stage renal disease (n=2136), do‐not‐resuscitate status or palliative care involvement (n=383), and cancer (n=1952) were excluded with reference to previous trials. Patients with missing data (n=12) were also excluded. Research participation status was identified using *ICD‐10‐CM* code Z00.6,^4^ which was restricted to code as the primary diagnosis or first secondary diagnosis to avoid overcapturing.

The primary outcome in this study was in‐hospital mortality. A multilevel logistic regression analysis accounting for strata and hospital clustering was performed to examine the association between clinical research participation status and in‐hospital mortality. A sensitivity analysis was performed in the propensity score‐matched cohort, which was generated by 1:1 nearest‐neighbor matching using a caliper width of 0.01 adjusting for baseline characteristics. Variables for these analyses were chosen on the basis of clinical relevance and the previous work^4^ and were included if they were significantly different in the baseline characteristics Table. Difference between groups were tested by 1‐way ANOVA and the chi‐square test, as appropriate. Statistical analysis was performed using Stata 16.1 (StataCorp) and R (R Foundation).

**Table:** Baseline Characteristics and In‐Hospital Outcomes in Clinical Research Participants and Nonparticipants Undergoing Transcatheter Aortic Valve Implantation

Characteristics	Before matching				After propensity score matching			
	Research participants (n=14 311)	Nonparticipants (n=37 072)	Overall (n=51 383)	*P* value	Research participants (n=14 301)	Nonparticipants (n=14 301)	Overall (n=28 602)	*P* value
Age, y	80.0±7.9	80.0±8.4	80.0±8.3	0.736	80.0±7.9	80.1±8.5	80.1±8.2	0.344
Female sex	46.0%	46.9%	46.6%	0.075	49.7%	50.3%	50.1%	0.281
Race or ethnicity				<0.001				<0.001
White	88.7%	87.7%	88.0%		89.5%	88.1%	88.8%	
Black	3.1%	3.9%	3.7%		2.9%	3.6%	3.2%	
Hispanic	4.2%	4.5%	4.4%		3.9%	4.2%	4.1%	
Asian	1.3%	1.2%	1.2%		1.2%	1.2%	1.2%	
Native American	0.3%	0.2%	0.3%		0.3%	0.2%	0.3%	
None of the above	2.4%	2.5%	2.4%		2.2%	2.8%	2.5%	
Insurance				<0.001				<0.001
Medicare	90.9%	89.2%	89.6%		90.9%	89.3%	90.1%	
Medicaid	0.8%	1.3%	1.2%		0.8%	1.2%	1.0%	
Private	6.6%	7.3%	7.1%		6.6%	7.4%	7.0%	
Self‐pay	0.5%	0.4%	0.5%		0.5%	0.5%	0.5%	
None of the above	1.2%	1.8%	1.6%		1.2%	1.6%	1.4%	
Median household income				<0.001				<0.001
$1–$38 999	18.7%	22.1%	21.1%		18.7%	20.6%	19.7%	
$39 000–$47 999	26.8%	25.0%	25.5%		26.8%	24.7%	25.8%	
$48 000–$62 999	28.9%	25.9%	26.7%		28.9%	25.9%	27.4%	
$63 000 or more	25.6%	27.1%	26.7%		25.6%	28.8%	27.2%	
Comorbidities								
Elixhauser comorbidity score				<0.001				0.975
0	0.03%	0.02%	0.02%		0.3%	0.3%	0.3%	
1–3	15.3%	12.1%	13.0%		15.3%	15.2%	15.2%	
4–5	38.5%	36.4%	37.0%		38.5%	38.8%	38.6%	
6 or more	46.2%	51.5%	50.0%		46.2%	46.0%	46.1%	
Hypertension	88.5%	88.2%	88.3%	0.400	88.5%	88.8%	88.7%	0.401
Diabetes	35.6%	36.6%	36.4%	0.037	35.6%	35.3%	35.5%	0.578
Obese	18.6%	18.6%	18.6%	0.947	18.6%	18.0%	18.3%	0.245
Chronic kidney disease without end‐stage renal disease	33.5%	32.9%	33.1%	0.246	33.5%	32.6%	33.0%	0.105
Anemia	4.5%	4.9%	4.8%	0.077	4.6%	4.3%	4.4%	0.261
Atrial fibrillation	37.6%	40.1%	39.4%	<0.001	37.6%	38.1%	37.9%	0.380
Congestive heart failure	71.2%	74.9%	73.9%	<0.001	71.3%	72.0%	71.7%	0.142
Arrhythmia	55.0%	58.6%	57.6%	<0.001	55.1%	55.2%	55.1%	0.831
Prior stroke	14.1%	14.3%	14.2%	0.537	14.1%	13.3%	13.7%	0.056
Prior myocardial infarction	11.9%	13.1%	12.8%	<0.001	11.9%	11.0%	11.5%	0.025
Prior percutaneous coronary intervention	21.6%	22.1%	21.9%	0.262	21.6%	20.7%	21.2%	0.066
Prior coronary artery bypass graft	17.0%	18.6%	18.2%	<0.001	17.0%	16.0%	16.5%	0.023
Prior PPM	9.7%	10.1%	10.0%	0.240	9.7%	9.3%	9.5%	0.219
Chronic pulmonary disease	28.4%	32.8%	31.6%	<0.001	28.4%	28.3%	28.3%	0.773
Pulmonary circulation disorders	16.7%	18.3%	17.9%	<0.001	16.7%	16.3%	16.5%	0.435
Peripheral vascular disease	22.2%	24.7%	24.0%	<0.001	22.2%	22.0%	22.1%	0.732
Liver disease without cirrhosis	1.8%	1.9%	1.9%	0.543	1.8%	1.5%	1.6%	0.051
Hypothyroidism	19.6%	20.5%	20.2%	0.024	19.6%	19.2%	19.4%	0.362
Hospital characteristics								
Hospital bed size				<0.001				<0.001
Small	7.4%	6.4%	6.6%		7.4%	5.9%	6.7%	
Medium	19.0%	20.4%	20.0%		19.0%	20.5%	19.7%	
Large	73.6%	73.2%	73.3%		73.6%	73.6%	73.6%	
Hospital region				<0.001				<0.001
Northeast	19.7%	25.4%	23.8%		19.7%	30.0%	24.8%	
Midwest	24.4%	22.3%	22.9%		24.4%	23.6%	24.0%	
South	29.8%	35.2%	33.7%		29.8%	32.4%	31.1%	
West	26.0%	17.1%	19.6%		26.0%	14.1%	20.1%	
Hospital location/teaching status				<0.001				<0.001
Rural	0.4%	1.2%	1.0%		0.4%	1.3%	0.8%	
Urban nonteaching	10.5%	8.9%	9.3%		10.5%	8.3%	9.4%	
Urban teaching	89.1%	89.9%	89.7%		89.1%	90.4%	89.8%	
In‐hospital outcomes								
All‐cause mortality	1.0%	1.5%	1.3%	<0.001	1.0%	1.4%	1.2%	0.002
Hospital discharge				<0.001				<0.001
Routine	68.4%	58.2%	61.1%		68.3%	58.7%	63.5%	
Transfer to short‐term hospital	0.2%	0.5%	0.4%		0.2%	0.5%	0.4%	
Skill nursing facility	12.5%	16.3%	15.2%		12.5%	15.6%	14.1%	
Home health care	17.9%	23.5%	22.0%		17.9%	23.7%	20.8%	
PPM implantation	8.8%	10.6%	10.1%	<0.001	8.8%	10.4%	9.6%	<0.001
Acute myocardial infarction	1.7%	1.8%	1.8%	0.310	1.7%	1.8%	1.7%	0.556
Cardiac arrest	0.9%	1.4%	1.2%	<0.001	0.9%	1.2%	1.1%	0.009
Cardiogenic shock	1.7%	2.0%	1.9%	0.012	1.7%	1.7%	1.7%	0.853
Ventricular tachycardia	3.1%	3.4%	3.3%	0.062	3.1%	3.1%	3.1%	0.973
AKI	9.9%	12.1%	11.5%	<0.001	9.9%	11.6%	10.8%	<0.001
AKI leading to dialysis	0.5%	0.5%	0.5%	0.495	0.5%	0.5%	0.5%	0.679
Respiratory failure	1.8%	3.8%	3.2%	<0.001	1.3%	2.2%	1.7%	<0.001
Vasopressor use	1.9%	2.3%	2.2%	0.007	1.9%	2.3%	2.1%	0.014
Intra‐aortic balloon pump	0.5%	0.7%	0.6%	0.020	0.5%	0.6%	0.5%	0.286
Percutaneous ventricular assist device	0.2%	0.3%	0.3%	0.329	0.2%	0.2%	0.2%	0.901
Extracorporeal membrane oxygenation	0.2%	0.2%	0.2%	0.040	0.2%	0.2%	0.2%	0.257
Total inflation adjusted cost, US dollars	$54 420±$24 731	$53 728±$28 011	$53 902±$27 225	<0.001	$54 423±$24 735	$53 313±$26 054	$53 831±$25 452	0.002
Length of hospital stay, d	3.9±4.8	4.8±5.6	4.5±5.4	0.318	3.9±4.8	4.6±5.4	4.3±5.1	<0.001

Values are mean±SD or %. AKI indicates acute kidney injury; and PPM, permanent pacemaker.

Between 2013 and 2019, 51 383 patients undergoing TAVI met the inclusion criteria: 14 311 (28%) research participants and 37 072 (72%) nonparticipants. Baseline patient characteristics are shown in Table. Compared with nonparticipants, research participants were less likely to have atrial fibrillation, chronic pulmonary disease, history of myocardial infarction, and a higher Elixhauser comorbidity score.

Crude in‐hospital mortality after TAVI was lower in research participants than in nonparticipants (1.0% versus 1.5%, *P*<0.001). Regarding TAVI‐related complications, permanent pacemaker implantation, cardiac arrest, cardiogenic shock, acute kidney injury, respiratory failure, vasopressor requirement, and requiring mechanical circulatory support were less frequent in research participants than in nonparticipants. Furthermore, research participants had higher total inflation adjusted costs than nonparticipants.

Crude in‐hospital mortality after TAVI in research participants decreased from 4.4% in 2013 to 0.6% in 2019 (Cochran‐Armitage trend *P*<0.001). In nonparticipants, crude in‐hospital mortality after TAVI also decreased from 3.6% in 2013 to 1.0% in 2019 (Cochran‐Armitage trend *P*<0.001).

After adjustment for baseline characteristics, adjusted odds ratio of in‐hospital death was significantly lower in research participants than nonparticipants (odds ratio, 0.72; 95% CI, 0.60–0.88; *P*<0.001). The propensity score‐matched cohort gave similar results in terms of in‐hospital outcomes (Table).

There are several possible explanations for the significant relationship between research participation status and in‐hospital mortality after TAVI in this study. First, physicians or hospitals participating in clinical research may have high‐level experience and provide cutting‐edge care. Previous studies demonstrated an inverse volume‐outcome relationship of TAVI.^5^ High‐volume hospitals seem to offer more opportunity for research participation, which may result in better outcomes. Second, behavior change in operators and postoperative care attributable to research participation may contribute to differences in patient outcomes, that is, the Hawthorne effect.^2^ Third, high frailty scores associated with an increased risk of adverse outcomes may preclude research participation.

Several limitations should be acknowledged. First, details on individual trials were lacking, such as inclusion and exclusion criteria and the reason for research participation. The interest of researchers may not be limited to TAVI. Second, the Nationwide Inpatient Sample database did not contain information regarding echocardiographic parameters, the Society of Thoracic Surgeons risk scores, frailty scores, and medication use. The impact of research participation status on outcomes should be further evaluated in patients with similar risk scores and frailty.

In conclusion, although our findings are subject to unmeasured confounders and selection bias for patients with favorable characteristics,^3^ research participation could be a determinant of better short‐term outcomes after TAVI. Further studies are needed to investigate the effect of research participation on long‐term outcomes after TAVI.

## Sources of Funding

None.

## Disclosures

None.
